# Imaging Comparison between ^**18**^F-FDG-PET/CT and ^**18**^F-Flouroethyl Choline PET/CT in Rare Case of Thymus Carcinoma Exhibiting a Positive Choline Uptake

**DOI:** 10.1155/2013/464396

**Published:** 2013-09-19

**Authors:** Mustafa Takesh, Stefan Adams

**Affiliations:** ^1^Department of Nuclear Medicine, Heidelberg University Hospital, Im Neuenheimer Feld 400, 69120 Heidelberg, Germany; ^2^Department of Nuclear Medicine and Radiology, Knappschaft Hospital, 66280 Sulzbach, Germany

## Abstract

It is of great value by using PET imaging in oncology to recognize any atypical uptake not related to the underlying disease. That helps in avoiding the false positive finding and may contribute in extending the application range of used radiopharmaceuticals in further disorders. It is well known that radiolabeled choline is an essential PET tracer used currently in prostate cancer. The physiological choline distribution was described. Nevertheless there is still a lack of studies, which describe this distribution in young patients; given that the radiolabeled choline is generally being used in the field of prostate cancer. Whether the thymus exhibits normally a positive choline uptake or not is still unknown. In particular, it is known that the lymphocytes express high affinity of choline transporter and enzymes involving its metabolism. Some cases of thymus carcinoma exhibiting a positive choline had been reported in the literature, however, mostly using ^11^C-choline. We report a rare case of metastatic thymic carcinoma detected incidentally using ^18^F-choline-PET in a 78-year-old male patient referred with elevation of prostate specific antigen. Moreover we present a comparison pattern with ^18^F-FDG-PET modality, in which ^18^F-choline-PET was turned out to be superior in tumor delineation.

## 1. Introduction

The field of diagnostic nuclear medicine is wide and open for developing new applications. Radiolabeled choline was introduced as a good PET tracer in assessing the prostate cancer.

Either labeled with carbon (^11^C) or with fluoride (^18^F), the choline is considered widely as the favorable choice in prostate tumor; primary and recurrent tumor as well.

Owing to the short half-life of ^11^C, the appliance of ^11^C-labeled choline is restricted in centers equipped with cyclotron on site. F-18-labeled choline was considered as an alternative to ^11^C labeled choline nearly in all applications.

Schillaci et al. [1] described the physiological distribution of F-18 fluorethyl choline in 80 patients with prostate cancer. Normal uptake was observed in liver, pancreas, spleen, salivary, and lachrymal glands. Abnormal uptake not owing to PCA was found in 15 patients out of 80 (18.7%).

Using ^11^C-labeled choline, Fallanca et al. [2] described an unusual case of incidental detection of thymoma in addition to local recurrence in prostate cancer patient referred due to biochemical recurrence. On the other hand, Calabria et al. [3] reported in 2011 the first case of thymus tumor detected incidentally by fluoride labeled choline.

Generally in thymus tumors, it remains a big challenge by using ^18^F-FDG-PET to detect them among wide range of mediastinal tumors, since nearly all these tumors show a positive FDG uptake. This fact emphasizes the importance of researching for another diagnostic method specific for thymus cancer. In this setting, Shibata et al. [4] described the role of ^11^C-acetate PET for diagnosing the histological type of thymus tumor. 

We describe in this report a rare case of thymus carcinoma with pleura metastases detected incidentally using ^18^F-FECH-PET. Additionally, we offer a comparison model with ^18^F-FDG-PET.

This case highlights the potential role of ^18^F-FECH-PET in identifying the thymus tumor and raises the question about the possibility to nominate it to turn into a standard diagnostic method for thymus cancer besides its main use in prostate cancer diagnosis.

## 2. Case Presentation

A 78-year-old male patient was referred to our department due to an unexplained PSA elevation. He underwent ^18^F-FECH-PET to evaluate the prostate gland.

**Figure 1:**
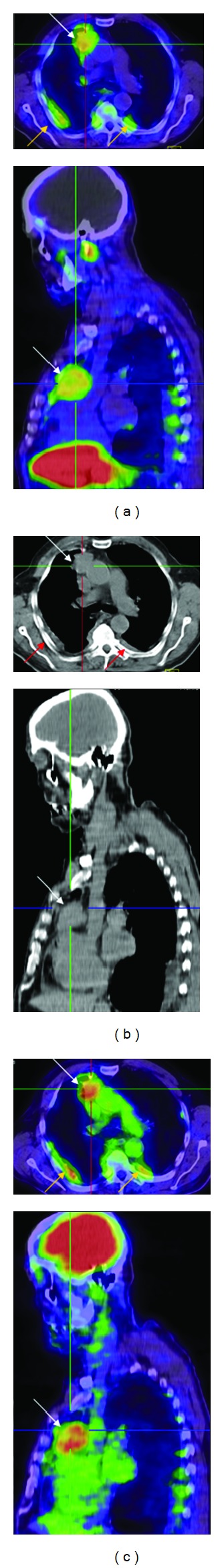
A 78-year-old male patient with biopsy confirmed thymic carcinoma. A comparison model between ^18^F-FECH-PET and ^18^F-FDG-PET. ^18^F-FECH-PET/CT fused imaging (a), ^18^F-FDG-PET/CT fused imaging (c) and matching CT imaging (b). Axial planes (upper row) and sagittal planes (lower row). The images demonstrate a thymic carcinoma in anterior mediastinum (white arrows) with pleural involvement (red arrows). We note a superior tumor-to-background ratio in ^18^F-FECH-PET/CT imaging in comparison to ^18^F-FDG-PET/CT imaging.

No evidence of prostate cancer was detected. However, F-18 fluoroethy lcholine-PET/CT demonstrated an increased uptake in a mass in the anterior mediastinum, besides two other positive findings corresponding morphologically with pleura thicknesses (Figure 1). This mass was confirmed to be thymic carcinoma with pleural involvement.

Given that ^18^F-FDG-PET is a method of value in the mediastinal tumors, further examination using ^18^F-FDG-PET was done three days later.

No additional data had been reported and the images were similar to that using ^18^F-FECH-PET, however, with few discrepancies.

Clearly, the uptake of ^18^F-FDG was more pronounced than choline uptake, that was also confirmed by SUV measurement (4, 08 in ^18^F-FECH-PET versus. 6, 05 in ^18^F-FDG-PET). However, by using ^18^F-FECH-PET the thymic tumor was the sole structure in mediastinum showing a positive uptake, which resulted in a high target to background signal ratio and better tumor delineation.

In contrast, by using ^18^F-FDG-PET, the mediastinal blood pool activity affects negatively the tumor demarcation.

Of course, the ^18^F-FECH-PET is not an established diagnostic tool for thymic cancer. Nevertheless such cases pave the way for further extended studies involving the potential benefit of ^18^F-FECH-PET in thymic cancer.

In conclusion, this report does not only display a rare case of thymic cancer detected incidentally by ^18^F-FECH-PET but provides an ideal comparison model between ^18^F-FECH-PET and ^18^F-FDG-PET in this uncommon tumor.

## 3. Discussion

### 3.1. Overview about Thymus and Relation with Cholinergic System

It is well know that the thymus is a specialized organ of the immune system. Its task is production and education of T-lymphocytes. Lymphocytes express most components of the cholinergic system including acetylcholine, muscarinic, and nicotinic acetylcholine receptors, choline acetyltransferase, and acetylcholinesterase [5, 6].

Based on this fact, normal thymus tissue is supposed to be of positive choline uptake.

While a physiologic ^18^F-FDG uptake in thymus has been reported in children and in sporadic cases in adults after chemotherapy [7], there is no report about physiologic ^18^F-FECH uptake in thymus. However, the pattern of physiological distribution of radiolabeled choline is not available in children, simply because its application is mostly in adult patients with prostate cancer.

### 3.2. Types of Thymus Tumors and Diagnostic Methods

Thymomas and thymic carcinomas are the most common tumors of the anterior mediastinum. Thymomas are generally encapsulated and have a mild histologic appearance. By contrast, thymic carcinomas present more likely in an invasive shape.

The complete resection improves survival in locally invasive thymic tumors.

Adjuvant postoperative radiation therapy may improve the outcome in patients with invasive disease [8].

Long term followup is required essentially concerning the incidence of local relapse. At this point in such critical anatomical location (anterior mediastinum), the morphological images are of negligible value in distinction between recurrent disease from postoperative or post-irradiation fibrosis.

Thus, there is a need of new nonanatomical methods able to recognize the residual tissue from surrounding tissue.

El-Bawab et al. [9] explained the importance of ^18^F-FDG-PET and its superiority over computed tomography during followup after thymoma excision, in the early detection and localization of meditational recurrence.

On the other hand, ^18^F-FECH-PET was demonstrated as a method of value in tumors with low glucose metabolism. There are enough studies describing its role in the diagnosis of prostate cancer. Thymic carcinoma has been already shown likely to have a positive uptake in ^18^F-FECH-PET; however, such report of metastatic thymus carcinoma detected using ^18^F-FECH-PET additionally with a comparison pattern with ^18^F-FDG-PET imaging was rare reported. In conclusion in the absence of the data describing a physiological ^18^F-FECH uptake in thymus gland, each uptake in thymus should be suspected in until proven otherwise.
